# IFN-β: A Contentious Player in Host–Pathogen Interaction in Tuberculosis

**DOI:** 10.3390/ijms18122725

**Published:** 2017-12-16

**Authors:** Naveed Sabir, Tariq Hussain, Syed Zahid Ali Shah, Deming Zhao, Xiangmei Zhou

**Affiliations:** State Key Laboratories for Agrobiotechnology, Key Laboratory of Animal Epidemiology of the Ministry of Agriculture, National Animal Transmissible Spongiform Encephalopathy Laboratory, College of Veterinary Medicine, China Agricultural University, Beijing 100193, China; naveedsabir@upr.edu.pk (N.S.); drtariq@aup.edu.pk (T.H.); zahidvet@cau.edu.cn (S.Z.A.S.); zhaodm@cau.edu.cn (D.Z.)

**Keywords:** *Mycobacterium tuberculosis* (Mtb), interferon-β (IFN-β), nucleotide-binding domain and leucine-rich repeat protein-3 (NLRP3), interleukin-1β (IL-1β), fate of infection

## Abstract

Tuberculosis (TB) is a major health threat to the human population worldwide. The etiology of the disease is *Mycobacterium tuberculosis* (Mtb), a highly successful intracellular pathogen. It has the ability to manipulate the host immune response and to make the intracellular environment suitable for its survival. Many studies have addressed the interactions between the bacteria and the host immune cells as involving many immune mediators and other cellular players. Interferon-β (IFN-β) signaling is crucial for inducing the host innate immune response and it is an important determinant in the fate of mycobacterial infection. The role of IFN-β in protection against viral infections is well established and has been studied for decades, but its role in mycobacterial infections remains much more complicated and debatable. The involvement of IFN-β in immune evasion mechanisms adopted by Mtb has been an important area of investigation in recent years. These advances have widened our understanding of the pro-bacterial role of IFN-β in host–pathogen interactions. This pro-bacterial activity of IFN-β appears to be correlated with its anti-inflammatory characteristics, primarily by antagonizing the production and function of interleukin 1β (IL-1β) and interleukin 18 (IL-18) through increased interleukin 10 (IL-10) production and by inhibiting the nucleotide-binding domain and leucine-rich repeat protein-3 (NLRP3) inflammasome. Furthermore, it also fails to provoke a proper T helper 1 (Th1) response and reduces the expression of major histocompatibility complex II (MHC-II) and interferon-γ receptors (IFNGRs). Here we will review some studies to provide a paradigm for the induction, regulation, and role of IFN-β in mycobacterial infection. Indeed, recent studies suggest that IFN-β plays a role in Mtb survival in host cells and its downregulation may be a useful therapeutic strategy to control Mtb infection.

## 1. Introduction

Tuberculosis remains a major global health threat to both human and animal populations. The disease is caused by *Mycobacterium tuberculosis* complex (MTBC), a group of highly related intracellular pathogens, and is a major cause of human morbidity and mortality worldwide [1]. Current estimations show that one-third of the world’s population is latently infected with Mtb. However, only 5–10% of infected people develop active tuberculosis (TB) in their lifetime [2]. It is the leading infectious cause of deaths, exceeding HIV/AID, and resulted in approximately 1.3 million human deaths in 2016 [3]. Host innate immune response is one of the most important determinants in the outcome of Mtb infection. The possible outcomes of an infection by a pathogen depend upon a complex interplay between virulence factors produced by the invading microbe and the immune responses mounted by the host defense system. These immune responses are characterized by an upregulation of many cytokines, chemokines, and products having direct or indirect antimicrobial activity [4,5]. However, many pathogens, for their survival, have evolved strategies to suppress and misdirect their host’s immune responses [6,7,8,9,10]. In this regard, Mtb has evolved multiple mechanisms for eluding or suppressing the host cell immune response and thus has become a successful intracellular pathogen.

Several cytokines have been shown to participate in the innate host response against Mtb, where they function either to enhance host resistance or aggravate the infection [11]. The critical role of inflammatory cytokines such as IL-1β has been demonstrated in the control of Mtb activity because the cytokines increase the antimicrobial function of macrophages [12]. On the other hand, IFN-β, an important cytokine, has been reported to have pro-bacterial activity and in many studies with animal models and humans has been associated with the development of TB [13,14,15,16]. Interferons (IFNs) have three major types: type I, II, and III. IFN-β is a member of type I IFN, a family of structurally related cytokines in humans and mice that includes several IFN-α (represented by several partially homologous genes) and one IFN-β (represented by a single gene) [17]. On the other hand, there is only one type II IFN, known as IFN-γ [18,19,20]. Type III IFN is the most recently discovered member of the IFN family [21,22,23,24].

IFN-β is a pleiotropic cytokine that is produced in response to a variety of pathogens including viruses, bacteria, and protozoa [25,26,27,28], and plays a crucial role in the stimulation of the innate and adaptive immune responses [29,30]. In general, type I IFN signals through type I IFN receptor (IFNAR), which consists of two subunits: IFN receptor 1 (IFNAR1) and IFN receptor 2 (IFNAR2) [31]. IFNAR depends on Janus kinase 1 (Jak1) and tyrosine kinase 2 (Tyk2) to phosphorylate signal transducer and activator of transcription 1 (STAT1) and 2 (STAT2) [32]. Phosphorylated STAT1 and STAT2 then lead to the formation of the transcriptional complex IFN-stimulated gene factor-3 (ISGF3) complex that translocates into the nucleus to induce gene expression [33]. Another transcriptional complex, the STAT1/STAT2 heterodimer, results in transcription of *ISG15*, *ISG54*, and *IFI6* genes [34,35]. These transcription factor functions are regulated by interferon regulatory factor 2 (IRF-2) and arginine methylation of STAT1 [36,37,38]. On the other hand, negative regulatory factors limiting type I IFN-mediated signaling include suppressor of cytokine signaling 1 (SOCS1), protein tyrosine phosphatase non-receptor type 6 (PTPN6), and ubiquitin-specific peptidase (UBP) [39,40,41,42,43].

Induction and regulation of IFN-β in Mtb infection is complex and has been vastly studied in recent years. In contrast to the established protective role of IFN-β in anti-viral immunity, it has been demonstrated that Mtb has evolved certain mechanisms to use IFN-β to enhance its intracellular survival [8,9]. Recent studies have shown that this pro-bacterial activity of IFN-β is correlated with its anti-inflammatory properties [13,44]. This is because IFN-β antagonizes the production and function of IL-1β and IL-18 through increased IL-10 production and inhibits the NLRP3 inflammasome [45]. This reduced IL-1β secretion is correlated with increased host susceptibility in Mtb infection [46,47]. IFN-β may also indirectly suppress the activation of the NLRP3 inflammasome via induction of interleukin 27 (IL-27) [48,49]. Similarly, type I IFN production was associated with reduced secretion of IL-12 and TNFα in both *L. monocytogenes* and *M. tuberculosis* infection models [50]. Therefore, IL-1 inhibition by type I IFNs can impair host resistance in TB [51,52]. Furthermore, IFN-β fails to initiate appropriate Th1 response with reduced expression of the MHC-II and IFNGR [53]. The general role of type I IFN as an immune regulator has been previously reviewed [54], so this review will focus on the detailed interplay between IFN-β and Mtb infection, including induction and regulation of IFN-β and its role in the fate of infection. Indeed, recent developments suggest that IFN-β plays a role in Mtb survival in host cells and is detrimental to host immunity. Therefore, its downregulation in Mtb infection may be a useful therapeutic approach for better control and treatment of TB.

## 2. Induction and Regulation of interferon-β (IFN-β) in Tuberculosis

Induction of IFN-β by Mtb and other members of the MTBC group is an important step for activation of innate immunity. This process is mediated by the activation of pattern-recognition receptors (PRR) such as Toll-like receptors (TLR) and cytosolic receptors such as retinoid-inducible gene 1 (RIG-I) and melanoma differentiation-associated gene 5 (MDA5) [55,56,57]. Infection of various human and mice cell types with Mtb activates interferon regulatory factors (IRFs) like IRF-3, IRF-5, and nuclear factor-κB (NF-κB), and leads to the expression of IFN-β [58,59,60,61]. IFN-β induction is mainly controlled at the level of gene transcription wherein a family of transcription factors and interferon regulatory factors (IRFs) play a central role [62]. Several studies on IRFs have provided new paradigms of how genes are ingeniously regulated during immune responses [59,63].

A comparison of gene expression profiles in mouse macrophages infected with virulent mycobacterial strains versus an avirulent mutant with an inactive early secretory antigenic target 6 (ESAT-6) system 1 (ESX-1) revealed the selective induction of IFN-β-associated genes by virulent, but not the avirulent, bacteria [64]. This virulence-associated IFN-β response was found to be independent of the TLR adaptor TIR-domain-containing adapter-inducing interferon-β (TRIF) and the receptor interacting protein-2 (RIP-2) but required Tank-binding kinase 1 (TBK1), a kinase also necessary for IFN-β induction by invading viruses and bacteria [15]. While partly confirming the above results, another report disagreed [65] and described that N-acetyl muramyl dipeptide 1 (Nod1), Nod2, and RIP-2 are required for IFN-β induction by virulent mycobacterial strains. The authors suggested that the role of ESX-1 in virulence determination is to facilitate bacterial Nod ligands getting in close contact with these host cytoplasmic receptors. It is tempting to assume that Mtb has evolved some mechanisms to use its own chromosomal DNA to provoke IFN-β response, as these cytokines are not only produced during in vivo infections [66] but also in active human disease [13]. Wassermann et al. [67] demonstrated that cyclic GMP–AMP synthase (cGAS) is an innate mycobacterial DNA sensor and have also provided some insight into how ESX-1 controls the activation of specific intracellular recognition pathways that lead to IFN-β and IL-1β production. Many other studies [68,69] have reported that cGAS is required for activating IFN-β production via the stimulator of interferon genes (STING)/TBK1/IRF3 pathway during Mtb and *Legionella pneumophila* infection of macrophages. Upon sensing cytosolic DNA, cGAS also activates cell-intrinsic antibacterial defenses, leading to an increased autophagic response against Mtb. Other researchers [15,70] have also reported that IFN-β response against Mtb requires ESX-1 activation, which contributes to the pathogenesis of the disease.

Previous investigations have shown indirect evidence implicating cytosolic mycobacterial DNA in triggering these IFN-β inducing pathways [67,70]. However, in a very recent study, Wiens et al. [71] described the role of mitochondrial dynamics and reported that host mitochondrial DNA (mtDNA), not mycobacterial DNA, contributes to IFN-β induction. Different strains of MTBC differentially induced IFN-β, though the strains did not differ in their access to the host cytosol and IFN-β induction by each strain required simultaneous stimulation of STING and cGAS. Treating macrophages with a mitochondria-specific antioxidant resulted in a reduced level of cytosolic mtDNA and inhibition of IFN-β induction by some strains. Furthermore, the variation in the role of mtDNA in IFN-β induction by different MTBC strains suggest that some additional mechanisms are also responsible for IFN-β signaling in Mtb infection. In addition, we have unveiled that another DNA sensor of interferon-inducible protein 204 (IFI204) plays an important role in IFN-β release in macrophages exposed to *M. bovis* [72].

Besides this, some studies [68,73] support a model in which Mtb triggers the STING/TBK1 pathway using the ESX-1 production system to interrupt phagosomal membranes and thereby allowing bacterial DNA access to cGAS in the cytosol. These results reveal that the mechanism of IFN-β induction in mycobacterial infection is more complex than the already established models suggest. Although the in vivo situation is certainly much more complex and the possible role of additional DNA sensors (which may work in close coordination with cGAS to activate the innate immune response) still remains unclear, it is believed that cGAS is a major player in the IFN-β signature associated with active TB [13,47].

## 3. IFN-β Regulating Signaling Pathways

### 3.1. IRF3 Pathway

The induction of type I IFN is controlled mainly at the transcriptional level, wherein a group of transcription factors known as interferon regulatory factors (IRFs) plays a pivotal role. Studies on most of the genes that encode IRFs have shown that IRFs have distinctive roles in the maturation and function of immune cells [62,74]. This IRF family of transcription factors comprises nine members, named consecutively from IRF1 to IRF9 [75,76]. Out of these nine IRF members, IRF3 and IRF7, which are structurally highly homologous, have gained much more attention as key regulators of type I IFN gene expression. It has been previously reported that IRF3 is a transcriptional factor responsible for stimulation of the IFN-β gene. IRF3 resides and is constitutively expressed in its inactive form. It undergoes phosphorylation and nuclear translocation upon viral and bacterial infection [77,78]. Although IRF7 also forms a homodimer or a heterodimer with IRF3 and acts on type I *IFN* gene family members, IRF3 is more potent in mediating the *IFN-β* gene, whereas IRF7 activates type I *IFN* genes [79,80].

The requirement of IRF3 and IRF7 transcription for IFN-β induction has been widely studied [81,82]. In early studies, IRF3 was reported to be responsible for the initiation of *IFN-β* gene induction by signals that induce the cooperative binding of IRF3 with other transcription factors; namely NF-κB and c-Jun/ATF-2 to the IFN-β promoter. This theory is supported by several lines of evidence [83]. It has also been described that *IFN-α* gene induction is affected in murine embryonic fibroblasts (MEFs) from mice deficient in IFN-β [84]. These results suggest that type I *IFN* gene induction occurs sequentially, wherein the initial IFN-β induction by IRF3 mediates the positive-feedback regulation of the gene induction mediated by the IFN-inducible IRF7 that can activate type I *IFN* genes. Unlike IRF3, IRF7 is downregulated in most cell types but it is strongly mediated by type I IFN signaling [80,81]. Stockinger et al. [85] reported that the cytosolic recognition of bacterial DNA results in IFN-β gene activation through the TBK1–IRF3 pathway, as proved by the absence of IFN-β induction in macrophages from TBK1^−^/^−^ or IRF3^−^/^−^ mice. In another study, it was shown that *L. monocytogenes* extracts that were pretreated with DNAase had an impaired ability to induce IFN-β, indicating an important role of cytosolic bacterial DNA in *IFN-β* gene induction [30]. Furthermore, the transfection of cells with dsDNA derived from either a pathogen or a host has been reported to induce type I IFN genes as well as many IFN-inducible genes via a TLR-independent mechanism [86,87]. This type I *IFN* gene induction mechanism requires TBK1 as well as IRF3. IRF3 is activated in response to lipopolysaccharides (LPS) in a MyD88 (myeloid differentiation primary response protein 88)-independent manner. It is involved in the LPS-induced MyD88-independent pathway and activated by the TLR3 ligand [88,89]. It is known that after stimulation with TLR4 and TLR3 ligands, LPS and dsRNA can induce IFN-β expression and subsequently lead to the induction of a set of IFN-inducible genes independently of MyD88, indicating that another adaptor protein may have a pivotal role in inducing IFN-β expression [90,91].

### 3.2. IFN-Stimulated Gene Factor-3 (ISGF3) Complex Pathway

An essential transcriptional complex is ISGF3. IFN-α/β binds to IFNAR1 and IFNAR2 and signals through receptor-bound Janus protein tyrosine kinases and STATs [31,32]. Activated STAT1/STAT2 link with interferon regulatory factor 9 (IRF9) to form ISGF3, which binds to IFN-stimulated response elements (ISREs) and upregulates IFN-β-stimulated genes (*ISGs*) [18]. These ISREs are found in the promoters of certain genes such as promyelocytic leukemia (PML), ISG15 ubiquitin-like modifier, IFN-induced protein with tetratricopeptide repeat 2, and IFN-α-inducible protein 6 (IFI6) [92,93,94]. This mature ISGF3 complex does not undergo further tyrosine phosphorylation, and is responsible for the activation of the *IRF7* gene. Similar to IRF3, IRF7 resides in the cytosol and, on activation, undergoes serine phosphorylation in its C-terminal region allowing its dimerization and nuclear translocation. IRF7 forms a homodimer or a heterodimer with IRF3 and each of these different dimers differentially act on the type I *IFN* gene family. Due to its susceptibility to ubiquitin-dependent degradation, IRF7 has a very short half-life (0.5–1 h) [95]. This labile nature of IRF7 represents a possible mechanism to control the *IFN* gene induction process to inhibit the overexpression of IFNs that may be injurious to the host.

### 3.3. NF-κB Pathway

NF-κB is a heterodimeric protein composed of different combinations of members of the Rel family of transcription factors. The Rel/ NF-κB family of transcription factors is involved mainly in stress-induced, immune, and inflammatory responses [96]. NF-κB is also an important regulator in cell fate decisions, such as programmed cell death and proliferation control, and it is critical in tumorigenesis [97]. The recognition of bacterial and viral products by TLRs on cells of the innate immune system also results in NF-κB induction, leading to the production of pro-inflammatory cytokines and the activation of antigen presenting cells (APCs). Type I IFN expression can be mediated by pathogens through endosomal membrane-bound TLRs, including TLR3, TLR7/8, and TLR9 [98,99]. Through MyD88 or TRIF adaptors, TLRs activate the kinases NF-κB, TBK1, and inducible IκB kinase (IKK) [100,101]. These kinases become phosphorylated and lead to activation of IRF3 and IRF7, which are critical for the induction of type I IFN [100]. IRF3 is constitutively expressed and plays a role in IFN-β expression following activation-induced dimer formation, while IRF7 expression is mediated through IFN feed-forward signaling and it is crucial for type I IFN expression [79,80].

**Figure 1:**
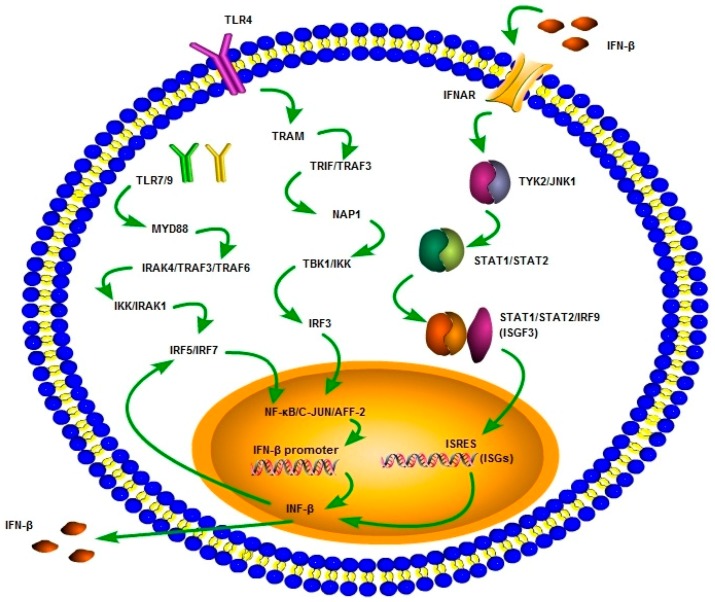
Interferon-β (IFN-β) signaling pathways and autocrine/paracrine loop. IFN-β signaling pathways may be MyD88-independent (TRIF or TLR3-4 pathways) or MyD88-dependent pathways. The pathway that is mainly triggered by TLR3 and TLR4 requires the adaptor protein TRIF, which leads to the production of type I IFNs. TRIF engages TNF receptor associated factor 3 (TRAF3) and NAP-1 (nucleosome assembly protein-1) to activate TBK1 and IKK. TBK1 and IKK lead to the induction of the transcription factor IRF3 inducing type I IFNs. On the other hand, stimulation of TLR7/8 or TLR9 leads to recruitment of MyD88 protein, IRAK4), TRAF3, and TRAF6 and results in activation of IRAK1, IKK-α, and transcription factors IRF5/IRF7, leading to the induction of type I IFNs. Once produced, IFN-β can also enhance the activation of IRF5/IRF7, leading to augmented type I IFN induction. In addition to these main pathways associated with the production of type I IFNs, there is also an autocrine/paracrine feedback loop amplifying the production of type I IFNs. This feedback loop is initiated by type I IFNs and leads to the formation of the ISGF3 complex. Binding of this ISGF3 complex to the IFN-stimulated response element (ISRE) within the ISRE-containing genes results in type I IFN induction.

The promoter of IFN-β contains NF-κB binding sites and two ISREs recognized by phosphorylated IRF3/7 [102]. Previous studies have shown that the activity of cooperating regulatory proteins recruited to DNA binding transcription factors plays an important role in the regulation of gene expression [103,104]. It was demonstrated that activation of IP10 (interferon-inducible protein-10), but not the MCP-1 promoter, both of which contain NF-κB binding sites differing in one and two nucleotides, requires IRF3 as a co-activator following LPS stimulation [105]. This suggests that the binding site sequence composition has an influence on the type of cooperative proteins that are recruited to complex with the NF-κB dimer. More recently, it has also been shown that glucocorticoid receptors can selectively trans-repress the transcription of a subset of genes (such as *Scyb9*) with promoters that use IRF3 as an essential co-activator of NF-κB binding upon LPS stimulation [106]. Besides these signaling pathways, an autocrine/paracrine feedback loop is also present, augmenting the induction of type I IFNs (Figure 1). This feedback loop is instigated by type I IFNs and leads to ISGF3 complex formation. The binding of this ISGF3 complex to the ISREs in the ISRE-containing genes results in amplified type I IFN production [107].

## 4. The Role of IFN-β in Tuberculosis

IFN-β signaling is crucial for host resistance against different pathogens [108]. Several Mtb-induced genes have key transcription factor binding sites for STATs, IRF-1, and IRF-7 leading to activation of the innate immune response [66]. Like other types of interferons, IFN-β has a ubiquitously expressed heterodimeric receptor composed of two chains, IFNAR1 and IFNAR2, which signal through Tyk2 and Jak1. This results in recruitment of STAT1 to receptor-bound STAT2 and production of STAT1–STAT2 heterodimers that detach from the receptors and migrate into the nucleus, leading to the activation of transcriptional factors [27]. Recently, it has been reported that IFN-β can signal through IFNAR1 independent of IFNAR2, and it is able to initiate a non-canonical signaling mechanism that controls the expression of a distinct set of genes [109]. IFN-β is capable of inducing many pathways in almost all cell types including immune cells where, on the one hand, it leads to increased secretion of certain cytokines such as interleukin-10 (IL-10) and interleukin-6 (IL-6) and, on the other hand, to blocked production and/or function of others like interleukin-17 (IL-17), interleukin-1 (IL-1), and IFN-γ [14].

The role of IFN-β has been well established in viral infections and hundreds of IFN-β-stimulated genes (*ISGs*) have been reported to have direct or indirect participation in antiviral immunity [110,111,112,113,114,115,116]. However, its protective or deleterious role in Mtb infection is much more complex and debatable [14]. As reported in another study, the continuous infusion of IFN-β to mice infected with *M. avium* resulted in increased resistance, as evidenced by a ten-fold reduction in hepatic and splenic bacterial loads [117]. However, in recent studies on Mtb infection in animal models and humans, endogenous IFN-β led to an increase in bacterial load and a reduced survival rate of the host [11,13,14,15,44,68,118]. In addition, a related pathogen, *M. bovis*, was shown to enhance replication rates in macrophages pre-treated with IFN-β [119]. The same pro-bacterial activity of IFN-β has been reported in mice infected with *Listeria* [120]. Furthermore, hypervirulence of a mycobacterial strain has been linked with enhanced IFN-β production, which is associated with impaired Th1 immune responses [16,68,121]. Later, it was reported that IFN-β receptor–deficient mice, chronically infected with a variety of different mycobacterial strains, demonstrated significantly reduced bacterial loads as compared to wild-type animals, while Mtb infected mice treated with IFN-β showed aggravated lung pathology and mycobacterial burden [122]. Dorhoi et al. [123] also demonstrated that IFN-β triggers immunopathological responses in TB-susceptible mice by modulating lung phagocyte dynamics. They reported that mice lacking IFNAR1 were protected and experienced reduced death rates upon aerosol infection with Mtb.

In a very recent study, de Toledo-Pinto et al. [124] identified many genes involving type I IFN that are differentially expressed in *M. leprae*-infected primary human Schwann cells. The gene encoding 2′-5′ oligoadenylate synthase-like (OASL) showed the greatest upregulation and was also upregulated in *M. leprae* infected human macrophages and primary monocytes. OASL knockdown was associated with decreased viability of *M. leprae* occurring in parallel to upregulation of either antimicrobial peptide expression or autophagy levels. *M. leprae*-mediated OASL expression was dependent on cytosolic DNA sensing mediated by STING. The addition of *M. leprae* DNA enhanced nonpathogenic *M. bovis* Bacillus Calmette-Guérin (BCG) intracellular survival, downregulated antimicrobial peptide expression, and increased monocyte chemoattractant protein-1 (MCP-1) secretion. Bouchonnet et al. [119] evaluated the effects of type I IFN on mycobacterial growth in human macrophages in vitro. They reported that type I IFN impairs the ability of human macrophages to control the growth of *M. bovis* BCG. Exogenous type I IFN resulted in increased mycobacterial growth because type I IFN has direct pleiotropic effects on the differentiation and functional activities of macrophages. These results indicate that type I IFN could directly stimulate mycobacterial growth in patients harboring these organisms. In another study, Auerbuch et al. [50] reported that mice lacking IFNAR1 are resistant to *L. monocytogenes* as compared to wild-type mice. Mariotti et al. [125] found that Mtb diverts type I IFN-induced monocyte differentiation from dendritic cells (DCs) into immuno-privileged macrophages. They reported that in the presence of type I IFN, Mtb might impede the renewal of potent antigen presenting cells (APCs) such as DCs, generating a safe habitat for intracellular growth. The study further suggested that Mtb has the ability to interfere specifically with monocyte differentiation. This ability may represent an effective Mtb strategy for eluding immune surveillance and persisting in the host.

**Figure 2:**
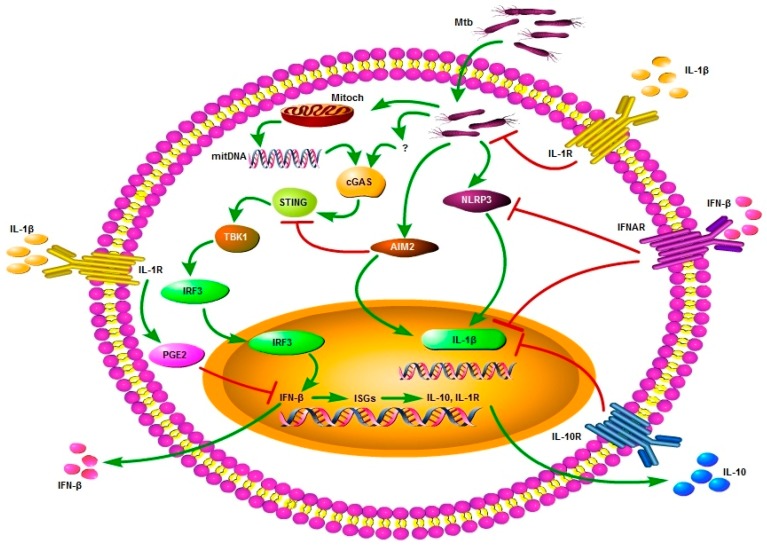
Role of IFN-β in host–pathogen interaction in Mtb infection. Mtb gains access to the host cytosol and leads to mitochondrial stress and release of mtDNA. This mtDNA and some other unknown factors indicated by “?” trigger the cGAS–STING–TBK1–IRF3–IFN-β pathway. On the other hand, Mtb also induces NLRP3 and AIM2 inflammasomes resulting in IL-1β synthesis. These two pathways have opposite roles and outcomes in the host defense against Mtb. In addition to direct inhibition of IL-1β, IFN-β can also inhibit or diminish the production of IL-1β through inhibition of NLRP3 inflammasomes and the augmented induction of IL-10. Reciprocal inhibition of IFN-β has been reported through IL-1β-induced PGE2. A recent study demonstrated that AIM2 could also inhibit IFN-β induction by interfering with the STNG–TBK1 pathway [72]. IL-1β is recognized as beneficial for host cells with anti-mycobacterial activity while IFN-β is considered largely detrimental for host cells having pro-bacterial and replication-promoting properties.

Taken together, the reports mentioned above show that, in contrast to the critical role played by IFN-β in anti-viral immune response, its induction during mycobacterial infection seems to be detrimental to the host by eluding and directing the host immune response towards a niche that permits and facilitates the intracellular survival of the pathogen (Figure 2). This pro-bacterial activity of IFN-β is related to its anti-inflammatory properties, not only because it antagonizes IL-1β’s production and function but also, in part, due to its failure to elicit a proper Th1 response and expression of MHC-II and interferon-γ receptors (IFNGRs) [121,122]. The pathways for regulating IFN-β in mycobacterial infection and the mechanism(s) by which it suppresses host resistance are currently active areas of investigation in the field of innate immunity.

Furthermore, many studies have demonstrated that type I IFNs can have an immunosuppressive role in the chronic phase of infection with *M. tuberculosis* and *M. leprae* in mice and humans [11,61,126]. Increased IL-10-mediated type I IFNs also contribute to the exacerbation of disease in a mouse model of *M. tuberculosis* infection [68]. Similarly, type I IFNs have primarily a suppressive role in chronic disseminated lepromatous leprosy lesions in patients infected with *M. leprae* [44]. Immunosuppressive pathways include for example the induction of IL-10 and programmed death-ligand 1 (PDL1), which antagonize the IFN-γ-induced antimicrobial response that drives pathogen clearance and disease resolution in self-healing tuberculoid lesions [44,127]. These studies provide a roadmap for future investigations on type I IFN blocking therapy to enhance specific immunity and facilitate clearance of chronic infections.

### 4.1. Immune Regulation by IFN-β in Tuberculosis

After its induction, type I IFN stimulates the formation of STAT-1 homodimers and ISGF3 [32,33]. In active Mtb infection, blood-based profiling has identified many genes induced by IFN-β [13]. It has been suggested that the expression of these immunologically important genes in Mtb-infected macrophages is independent of both TLR2 and TLR4, but it is dependent on IFNAR and STAT1 [128]. Results presented in another study indicate that production of host-protective cytokines such as tumor necrosis factor α (TNF-α), IL-12, and IL-1β is inhibited by exogenous type I IFN, whereas production of immunosuppressive IL-10 is promoted in an IL-27–independent manner [129]. Furthermore, much of the ability of type I IFN to inhibit cytokine production is mediated by IL-10, corroborating the idea that these IFNs inhibit the immune response during tuberculosis. Antonelli and his colleagues [122] treated pathogen-exposed mice intra-nasally with polyinosinic–polycytidylic acid condensed with poly-l-lysine and carboxymethylcellulose (poly-ICLC), an agent designed to stimulate prolonged, high-level production of type I IFN. Drug-treated Mtb–infected wild-type (WT) mice, but not mice lacking IFNAR1, displayed marked elevations in lung bacillary loads, accompanied by widespread pulmonary necrosis without detectable impairment of Th1 effector function. The above findings suggest that poly-ICLC treatment detrimentally affects the outcome of Mtb infection by promoting the accumulation of a permissive myeloid population in the lung.

Nevertheless, activation of mycobacterial infection is not a recognized adverse effect associated with the therapeutic use of recombinant type I IFN. As reported in earlier studies, administration of aerosolized IFN-α to patients receiving antimicrobial treatment for pulmonary tuberculosis led to a more rapid decrease in the number of bacilli identified in sputum and earlier resolution of fever and some radiographic abnormalities [130,131]. These immune-modulatory roles of IFN-α may be responsible for this salutary effect. The net effect of type I IFN on immune and inflammatory responses in humans and in murine models has been, however, quite variable in different situations [132,133]. The administration of type I IFN to mice infected with Mtb appears to impair the development of Th1 responses [134], which is thought to be necessary for protection against mycobacterial infection. There are also some indications that type I IFN may exert negative effects on mycobacteriostatic activity. Several case reports describing the appearance of mycobacterial infections in patients receiving IFN-α have been published [135,136], and the administration of type I IFN to mice infected with Mtb has been shown to enhance mycobacterial growth [134]. The extent to which an increase in type I IFN production influences mycobacterial replication in vivo still requires further investigations. These findings suggest that agents that stimulate type I IFNs should be used with caution in patients with active mycobacterial disease. On the other hand, Prabhakar et al. [137] reported that Mtb could inhibit IFN-α signaling by blocking type I IFN stimulated tyrosine phosphorylation of STAT-1. Infection with *M. bovis* BCG does not inhibit type I IFN-stimulated tyrosine phosphorylation of STAT-1, formation of homodimers, or transcription of genes regulated by STAT-1 homodimers, suggesting that inhibition of the response to type I IFN is related to the pathogenicity of Mtb.

### 4.2. IFN-β Suppresses IL-1 Production and Inflammasome Activation

IL-1 is an important and well-known cytokine with antibacterial properties [12]. It plays a pivotal role in the induction of the inflammatory and immune response against virulent mycobacterial strains but it is suppressed by type I IFN [47,51]. IFN-β-induced inhibition of IL-1 production has been reported by Guarda et al. [45] and Ma et al. [138] through two distinct pathways. IFN-β signaling via the STAT1 transcription factor repressed the activity of NLRP1 and NLRP3 inflammasomes, hence suppressing caspase-1-dependent IL-1β maturation. In addition, IFN-β induced IL-10 in a STAT1-dependent manner, and then IL-10 via autocrine action led to reduced production of pro-IL-1α and pro-IL-1β through STAT3 signaling. Mayer-Barber et al. [47] reported that IFN-β inhibited IL-1 production by both subsets, while CD4^+^ T cell-derived IFN-γ suppressed IL-1 expression selectively in inflammatory monocytes. This data provided cellular evidence for the anti-inflammatory effects and pro-bacterial functions of IFN-β during mycobacterial infection. In another report by the same author [52], it was revealed that IL-1/prostaglandin E2 (PGE2) is another important pathway by which IFN-β antagonizes IL-1 production during mycobacterial infection. The absence of IFN-β signaling resulted in increased PGE2 and IL-1β and decreased IL1Ra. Mtb-infected wild-type bone marrow-derived macrophages (BMDM) produced significantly less PGE2 when exogenous IFN-β was present. Novikov et al. [47] demonstrated that IFN-β selectively limits the production of IL-1β. This regulation occurs at the level of IL-1β mRNA expression, rather than caspase-1 activation or autocrine IL-1 amplification, and this regulation is only evident with the virulent mycobacterial strains, as avirulent strains fail to trigger the same response. Reciprocal control of type I IFNs by the IL-1/PGE2-mediated pathway has also been reported, and PGE2 treatment led to reduced production of type I IFNs and increased protection against Mtb infection [28,52]. Briken et al. [139] reviewed the role of IFN-β after its induction by mycobacterial infection, showing it could suppress NLRP3-inflammasome activation while increasing the action of AIM2 (absent in melanoma 2) inflammasomes. The suppressive effects of IFN-β on NLRP3 inflammasome activation have been reported by other researchers [47,51].

AIM2-inflammasome induction by mycobacterial infection was demonstrated in a previous study in which non-tuberculous mycobacteria (NTM) such as *M. smegmatis* (Msme), but not virulent mycobacteria, were reported to induce AIM2 inflammasomes in an IFN-β-dependent manner [64]. Moreover, Mtb was able to inhibit AIM2 inflammasome activation induced by Msme or by transfected dsDNA depending upon the ESX-1 secretion system, because an ESX-1-deficient Mtb mutant failed to inhibit AIM2 activation. The ESX-1 system is also important for the escape of Mtb from autophagy [140,141]. In contrast, a recent study by our research group [72] reported that *M. bovis*-induced AIM2 inflammasome activation decreases autophagy in primary and immortalized cells. Furthermore, we showed that the AIM2 cytosolic DNA sensor may conjugate competitively with cytosolic *M. bovis* DNA to restrict *M. bovis*-induced STING–TBK1-dependent autophagy activation and IFN-β secretion.

The role of IFN-β signaling in inducing AIM2 inflammasomes has been reported for macrophages infected with *F. tularensis* and *L. monocytogenes* [142,143]. Some studies have revealed that IFN-β acts at the transcription and translation levels of AIM2 in order to enhance the AIM2 inflammasome activity [143,144]. However, in another report, researchers failed to detect changes in protein levels of AIM2 after infection with *F. tularensis* [145]. This enigma requires further investigation to determine the molecular mechanism of interaction among the ESX-1 system of virulent mycobacteria, the AIM2 inflammasome, and IFN-β signaling. Comparing Mtb with NTMs may help reveal the inhibitory abilities of Mtb with respect to host cell death [146] and host cell autophagy [140,141]. It can be inferred that Mtb has adapted the methods to exploit the pathogen-beneficial functions of IFN-β to aggravate the disease.

### 4.3. Cross-Talk between Type I and Type II IFNs in Mycobacterial Infection

IFN-γ-induced Th1 responses have a critical role against mycobacterial infection. Therefore, one of the suggested mechanisms for type I IFN-mediated loss of Mtb control is the inhibition of Th1 responses [121]. It is well known that type I IFNs induce the immunosuppressive cytokine IL-10, which leads to loss of protection against Mtb [129]. Similarly, in *M. leprae* infection of humans, an IL-10-mediated suppression of IFN-γ signaling by type I IFNs has been linked to the lepromatous rather than the better-controlled tuberculoid form of the disease [44]. This study revealed an inverse correlation between type I IFNs and *IFN-γ* gene expression, and suggested that the differential production of IFNs contributes to protection versus pathogenesis in mycobacterial infections. Another study depicted antagonistic effects of type I IFNs on IFN-γ and as a result increased host susceptibility to bacterial infections [147]. These results support a previous study [53] in which it was also demonstrated that type I IFNs increase host susceptibility to *L. monocytogenes* whereas IFN-γ activates macrophages to resist the infection. The studies described that these opposing immunological effects of type I IFNs and IFN-γ occur because of cross-talk between the respective signaling pathways. Such cross-talk leads to dominance of type I IFN immune responses and may play a role in the beneficial effects of IFN-γ treatment against inflammatory diseases.

### 4.4. Role of Type I IFNs in Mycobacterial and HIV Co-Infection

It has been established that viral infections are potent stimuli for the production of type I IFNs [148,149]. Some studies have proposed the idea that increased type I IFN production mediated by certain viral infections could contribute to the increased susceptibility of patients to TB. An increased growth rate of Mtb in HIV type 1 (HIV-1)-infected macrophages has been demonstrated, although the mechanism by which viral-induced type I IFNs led to increased susceptibility to tuberculosis has not been explored [150]. On the other hand, transcription from the long terminal repeat of HIV-1 is highly increased in macrophages from convalescent HIV-1-infected individuals with active mycobacterial infections, resulting in increased viral replication [151]. Thus, in the human population having combined infections with both pathogens, a malevolent cycle might be established that is beneficial for the replication of both mycobacteria and HIV-1.

## 5. Conclusions and Future Prospects

Tuberculosis is the second major cause of morbidity and mortality, after HIV, in the human population worldwide. Infection with Mtb leads to a complex host–pathogen interaction involving up- and downregulation of many inflammatory and immune-regulatory cytokines. IFN-β is one of the most important cytokines and the mechanism for its induction and function in mycobacterial infection is complex. Although some other factors remain undiscovered, it is believed that cGAS, an innate sensor of mycobacterial infection, is a major player contributing to the IFN-β signature associated with active disease in humans. The immune-suppressive and pro-bacterial roles of IFN-β are revealed by its ability to downregulate IL-1β and IL-18, and upregulate IL-10. IL-1β has a protective role against virulent mycobacterial strains and it is downregulated through distinct pathways. IFN-β signaling, via the STAT1 transcription factor, represses the activity of the NLRP1 and NLRP3 inflammasomes, leading to the suppression of caspase-1-dependent IL-1β maturation. In addition, IFN-β promotes IL-10 induction and inhibits IFN-γ- and IL-12-mediated anti-bacterial activity of immune modulating cells. The reciprocal inhibition of IFN-β is mediated through the IL-1–PGE2 pathway. AIM2-inflammasome signaling during infection by virulent and avirulent mycobacteria and its interaction with IFN-β has been reported, but the findings are not in accord with each other. Therefore, the interaction of the ESX-1 secretion system, IFN-β, and the AIM2 inflammasome in Mtb infection remains an important question for future investigations.

It can be concluded that in contrast to the pivotal role played by IFN-β in anti-viral immune response, its induction during mycobacterial infection seems to be injurious to the host. The above findings implicate IFN-β as a downregulator of protective immune responses in mycobacterial infection. Furthermore, recognition of the multiple points of innate immune regulation, including the cGAS–STING–TBK1–IRF3–IFN-β, NLRP3/AIM2/IL-1β, and IL-1β/PGE2/IFN-β pathways, provides potential therapeutic targets. We expect that the information presented in this review about the induction and regulation of IFN-β, and its subsequent role in immune modulation in mycobacterial infection, will be helpful for better understanding host–pathogen interactions in TB. Furthermore, it will also help in the design of future strategies to confine the intracellular activities of this deadly pathogen for better control and treatment of the disease.

## References

[1] Lechartier B., Rybniker J., Zumla A., Cole S.T. (2014). Tuberculosis drug discovery in the post-post-genomic era. EMBO Mol. Med..

[2] Bhatt K., Salgame P. (2007). Host innate immune response to *Mycobacterium tuberculosis*. J. Clin. Immunol..

[3] WHO Global Tuberculosis Report, 2017. http://www.who.int/tb/publications/global_report/en/.

[4] Chen Z., Tato C.M., Muul L., Laurence A., O’Shea J.J. (2007). Distinct regulation of interleukin-17 in human T helper lymphocytes. Arthritis Rheum..

[5] Wilson N.J., Boniface K., Chan J.R., McKenzie B.S., Blumenschein W.M., Mattson J.D., Basham B., Smith K., Chen T., Morel F. (2007). Development, cytokine profile and function of human interleukin 17-producing helper T cells. Nat. Immunol..

[6] Coombes B.K., Valdez Y., Finlay B.B. (2004). Evasive maneuvers by secreted bacterial proteins to avoid innate immune responses. Curr. Biol..

[7] Choy A., Dancourt J., Mugo B., O’Connor T.J., Isberg R.R., Melia T.J., Roy C.R. (2012). The Legionella effector RavZ inhibits host autophagy through irreversible Atg8 deconjugation. Science.

[8] Gao D., Wu J., Wu Y.T., Du F., Aroh C., Yan N., Sun L., Chen Z.J. (2013). Cyclic GMP-AMP Synthase is an innate immune sensor of HIV and other retroviruses. Science.

[9] Sun L., Wu J., Du F., Chen X., Chen Z.J. (2013). Cyclic GMP-AMP synthase is a cytosolic DNA sensor that activates the type I interferon pathway. Science.

[10] Tattoli I., Sorbara M.T., Yang C., Tooze S.A., Philpott D.J., Girardin S.E. (2013). *Listeria* phospholipases subvert host autophagic defenses by stalling pre-autophagosomal structures. EMBO J..

[11] O’ Garra A., Redford P.S., McNab F.W., Bloom C.I., Wilkinson R.J., Berry M.P. (2013). The immune response in tuberculosis. Annu. Rev. Immunol..

[12] Fremond C.M., Togbe D., Doz E., Rose S., Vasseur V., Maillet I., Jacobs M., Ryffel B., Quesniaux V.F. (2007). IL-1 receptor-mediated signal is an essential component of MyD88-dependent innate response to *Mycobacterium tuberculosis* infection. J. Immunol..

[13] Berry M.P., Graham C.M.F., McNab W., Xu Z., Bloch S.A., Oni T., Wilkinson K.A., Banchereau R., Skinner J., Wilkinson R.J. (2010). An interferon-inducible neutrophil driven blood transcriptional signature in human tuberculosis. Nature.

[14] Trinchieri G. (2010). Type I interferon: friend or foe?. J. Exp. Med..

[15] Stanley S.A., Johndrow J.E., Manzanillo P., Cox J.S. (2006). The type I IFN response to infection with *Mycobacterium tuberculosis* requires ESX-1-mediated secretion and contributes to pathogenesis. J. Immunol..

[16] Manca C., Tsenova L., Freeman S., Barczak A.K., Tovey M., Murray P.J., Barry C., Kaplan G. (2005). Hypervirulent *M. tuberculosis* W/Beijing strains upregulate type I IFNs and increase expression of negative regulators of the Jak-STAT pathway. J. Interferon Cytokine Res..

[17] Decker T., Müller M., Stockinger S. (2005). The yin and yang of type I interferon activity in bacterial infection. Nat. Rev. Immunol..

[18] Platanias L.C. (2005). Mechanisms of type I- and type II-interferon-mediated signaling. Nat. Rev. Immunol..

[19] Chen J., Baig E., Fish E.N. (2004). Diversity and relatedness among the type I interferons. J. Interferon Cytokine Res..

[20] De Weerd N.A., Samarajiwa S.A., Hertzog P.J. (2007). Type I interferon receptors: Biochemistry and biological functions. J. Biol. Chem..

[21] Sheppard P., Kindsvogel W., Xu W., Henderson K., Schlutsmeyer S., Whitmore T.E., Kuestner R., Garrigues U., Birks C., Roraback J. (2003). IL-28, IL-29 and their class II cytokine receptor IL-28R. Nat. Immunol..

[22] Fox B.A., Sheppard P.O., O’Hara P.J. (2009). The role of genomic data in the discovery, annotation and evolutionary interpretation of the interferon-λ family. PLoS ONE.

[23] Prokunina-Olsson L., Muchmore B., Tang W., Pfeiffer R.M., Park H., Dickensheets H., Hergott D., Porter-Gill P., Mumy A., Kohaar I. (2013). A variant upstream of *IFNL3* (*IL28B*) creating a new interferon gene *IFNL4* is associated with impaired clearance of hepatitis C virus. Nat. Genet..

[24] Dumoutier L., Tounsi A., Michiels T., Sommereyns C., Kotenko S.V., Renauld J.C. (2004). Role of the interleukin (IL)-28 receptor tyrosine residues for antiviral and antiproliferative activity of IL-29/interferon-λ1: Similarities with type I interferon signaling. J. Biol. Chem..

[25] Parmar S., Platanias L.C. (2003). Interferons: Mechanisms of action and clinical applications. Curr. Opin. Oncol..

[26] Bogdan C. (2000). The function of type I interferons in antimicrobial immunity. Curr. Opin. Immunol..

[27] Decker T., Stockinger S., Karaghiosoff M., Müller M., Kovarik P. (2002). Interferons and STATs in innate immunity to microorganisms. J. Clin. Investig..

[28] Xu X.J., Reichner J.S., Mastrofrancesco B., Henry W.L., Albina J.E. (2008). Prostaglandin E2 suppresses lipopolysaccharide stimulated IFN-β production. J. Immunol..

[29] Bon A.L., Thompson C., Durand E.K.V., Rossmann C., Kalinke U., David F. (2006). Cutting edge: Enhancement of antibody responses through direct stimulation of B and T cells by type I IFN. J. Immunol..

[30] Stetson D.B., Medzhitov R. (2006). Type I interferons in host defense. Immunity.

[31] Pestka S., Langer J.A., Zoon K.C., Samuel C.E. (1987). Interferons and their actions. Annu. Rev. Biochem..

[32] Ghoreschi K., Laurence A., O’Shea J.J. (2009). Janus kinases in immune cell signaling. Immunol. Rev..

[33] McComb S., Erin C., Alturki A.N., Joseph J., Shutinoski B., Startek J.B., Gamero A.M., Mossman K.L., Sad S. (2014). Type-I interferon signaling through ISGF3 complex is required for sustained RIP3 activation and necroptosis in macrophages. PNAS.

[34] Ghislain J.J., Wong T., Nguyen M., Fish E.N. (2001). The interferon-inducible STAT2: STAT1 heterodimer preferentially binds in vitro to a consensus element found in the promoters of a subset of interferon-stimulated genes. J. Interferon Cytokine Res..

[35] Brierley M.M., Fish E.N. (2005). Functional relevance of the conserved DNA-binding domain of STAT2. J. Biol. Chem..

[36] Masumi A., Ozato K. (2001). Coactivator p300 acetylates the interferon regulatory factor-2 in U937 cells following phorbol ester treatment. J. Biol. Chem..

[37] Meraro D., Gleit-Kielmanowicz M., Hauser H., Levi B.Z. (2002). IFN-stimulated gene 15 is synergistically activated through interactions between the myelocyte/lymphocyte-specific transcription factors, PU.1, IFN regulatory factor-8/IFN consensus sequence binding protein, and IFN regulatory factor-4: Characterization of a new subtype of IFN-stimulated response element. J. Immunol..

[38] Mowen K.A., Zhu J.W., Schurter B.T., Shuai K., Herschman H.R., David M. (2001). Methylation of STAT1 modulates IFNα/β-induced transcription. Cell.

[39] Fenner J.E., Starr R., Cornish A.L., Zhang J.G., Metcalf D., Schreiber R.D., Sheehan J., Hilton K.D., Alexander W.S., Hertzog P.J. (2006). Suppressor of cytokine signaling 1 regulates the immune response to infection by a unique inhibition of type I interferon activity. Nat. Immunol..

[40] David M., Chen H.E., Goelz S., Larner A.C., Neel B.G. (1995). Differential regulation of the α/β interferon-stimulated Jak/STAT pathway by the SH2 domain-containing tyrosine phosphatase SHPTP1. Mol. Cell Boil..

[41] You M., Yu D.H., Feng G.S. (1999). Shp-2 tyrosine phosphatase functions as a negative regulator of the interferon-stimulated Jak/STAT pathway. Mol. Cell Boil..

[42] Myers M.P., Andersen J.N., Cheng A., Tremblay M.L., Horvath C.M., Parisien J.P., Salmeen A., Barford D., Tonks N.K. (2001). TYK2 and Jak2 are substrates of protein-tyrosine phosphatase 1B. J. Boil. Chem..

[43] Malakhova O.A., Kim K.I., Luo J.K., Zou W., Kumar K.G., Fuchs S.Y., Shuai K., Zhang D.E. (2006). UBP43 is a novel regulator of interferon signaling independent of its ISG15 isopeptidase activity. EMBO J..

[44] Teles R.M., Krutzik T.G., Montoya S.R., Schenk D., Lee M., Komisopoulou D.J., Kelly-Scumpia E., Chun K., Iyer R., Sarno S.S. (2013). Type I interferon suppresses type II interferon-triggered human antimycobacterial responses. Science.

[45] Guarda G., Mraun B., Staehli F., Tardivel A., Mattmann C., Farlik M., Decker T., Du Pasquier R.A., Romero P., Tschopp J. (2011). Type I interferon inhibits interleukin-1 production and inflammasome activation. Immunity.

[46] Mayer-Barber K.D., Barber D.L., Shenderov K., White S.D., Wilson M.S., Cheever A. (2010). Caspase-1 independent IL-1β production is critical for host resistance to *Mycobacterium tuberculosis* and does not require TLR signaling in vivo. J. Immunol..

[47] Novikov A., Cardone M., Thompson R., Shenderov K., Kirschman K.D., Mayer-Barber K.D., Myers T.G., Rabin R.L., Trinchieri G., Sher A. (2011). *Mycobacterium tuberculosis* triggers host type I IFN signaling to regulate IL-1β production in human macrophages. J. Immunol..

[48] Molle C., Goldman M., Goriely S. (2010). Critical role of the IFN-stimulated gene factor 3 complex in TLR-mediated IL-27p28 gene expression revealing a two-step activation process. J. Immunol..

[49] Mascanfroni I.D., Yeste A., Vieira S.M., Burns E.J., Patel B., Sloma I., Wu Y., Mayo L., Ben-Hamo R., Efroni S. (2013). IL-27 acts on DCs to suppress the T cell response and autoimmunity by inducing expression of the immunoregulatory molecule CD39. Nat. Immunol..

[50] Auerbuch V., Dirk G., Meyer-Morse N., O’Riordan M., Daniel A. (2004). Mice lacking the type I interferon receptor are resistant to *Listeria monocytogene*. J. Exp. Med..

[51] Mayer-Barber K.D., Andrade B.B., Barber D.L., Hieny S., Feng C.G., Caspar P., Oland S., Gordon S., Sher A. (2011). Innate and adaptive interferons suppress IL-1a and IL-1b production by distinct pulmonary myeloid subsets during *Mycobacterium tuberculosis* infection. Immunity.

[52] Mayer-Barber K.D., Andrade B.B., Oland S.D., Amaral E.P., Barber D.L., Gonzales J., Derrick S.C., Shi R., Kumar N.P., Wei W. (2014). Host-directed therapy of tuberculosis based on interleukin-1 and type I interferon crosstalk. Nature.

[53] Rayamajhi M., Humann J., Penheiter K., Andreasen K., Lenz L.L. (2010). Induction of IFN-αβ enables *Listeria monocytogenes* to suppress macrophage activation by IFN-γ. J. Exp. Med..

[54] Biron C.A. (2001). Interferons α and β as immune regulators—A new look. Immunity.

[55] Cai X., Chiu Y.H., Chen Z.J. (2014). The cGAS-cGAMP-STING pathway of cytosolic DNA sensing and signaling. Mol. Cell.

[56] Kawai T., Akira S. (2011). Toll-like receptors and their crosstalk with other innate receptors in infection and immunity. Immunity.

[57] Loo Y.M., Gale M. (2011). Immune signaling by RIG-I-like receptors. Immunity.

[58] Ottenhoff T.H.M., Dass R.H., Yang N., Zhang M.M., Wong H.E.E. (2012). Genome-wide expression profiling identifies type 1 interferon response pathways in active tuberculosis. PLoS ONE.

[59] Steinhagen F., McFarland A.P., Rodriguez L.G., Tewary P., Jarret A., Savan R., Dennis M.K. (2013). IRF-5 and NF-κB p50 co-regulate IFN-β and IL-6 expression in TLR9-stimulated human plasmacytoid dendritic cells. Eur. J. Immunol..

[60] Schneider W.M., Chevillotte M.D., Rice C.M. (2014). Interferon-stimulated genes: A complex web of host defenses. Ann. Rev. Immunol..

[61] Teijaro J.R., Mee L.A., Sullivan B.M., Sheehan K.C., Welch M., Schreiber R.D., de la Torre J.C., Oldstone M.B. (2013). LCMV infection is controlled by blockade of type I interferon signaling. Science.

[62] Honda K., Taniguchi T. (2006). IRFs: Master regulators of signalling by Toll-like receptors and cytosolic pattern-recognition receptors. Nat. Rev. Immunol..

[63] Honda K., Takaoka A., Taniguchi T. (2006). Type I inteferon gene induction by the interferon regulatory factor family of transcription factors. Immunity.

[64] Shah S., Bohsali A., Ahlbrand S.E., Srinivasan L., Rathinam V.A., Vogel S.N., Fitzgerald K.A., Sutterwala F.S., Briken V. (2013). *Mycobacterium tuberculosis* but not non-virulent mycobacteria inhibit IFN-β and AIM2-inflammasome dependent IL-1β production via their ESX-1 secretion system. J. Immunol..

[65] Pandey A.K., Yang Y., Jiang Z., Fortune S.M., Coulombe F. (2009). NOD2, RIP2 and IRF5 Play a Critical Role in the Type I Interferon Response to *Mycobacterium tuberculosis*. PLoS Pathog..

[66] Manzanillo P.S., Shiloh M.U., Portnoy D.A., Cox J.S. (2012). *Mycobacterium tuberculosis* activates the DNA-dependent cytosolic surveillance pathway within macrophages. Cell Host Microbe.

[67] Wassermann R., Gulen M.F., Sala C., Perin S.G., Lou Y., Rybniker J., Schmid-Burgk J.L., Schmidt T., Hornung V., Cole S.T. (2015). *Mycobacterium tuberculosis* differentially activates cGAS- and inflammasome-dependent intracellular immune responses through ESX-1. Cell Host Microbe.

[68] Wu J., Sun L.J., Chen X., Du F.H., Shi H.P., Chen C., Zhijian J.C. (2013). Cyclic-GMP-AMP is an endogenous second messenger in innate immune signaling by cytosolic DNA. Science.

[69] Watson R.O., Bell S.L., MacDuff D., AKimmey J.M., Diner E.J., Olivas J., Vance R.E., Stallings C.L., Virgin H.W., Cox J.S. (2015). The cytosolic sensor cGAS detects *Mycobacterium tuberculosis* DNA to induce type I interferons and activate autophagy. Cell Host Microbe.

[70] McNab F.W., Ewbank J., O’Garra A. (2013). TPL-2-ERK1/2 signaling promotes host resistance against intracellular bacterial infection by negative regulation of type I IFN production. J. Immunol..

[71] Wiens K.E., Ernst J.D. (2016). The Mechanism for Type I Interferon Induction by *Mycobacterium tuberculosis* is Bacterial Strain-Dependent. PLoS Pathog..

[72] Liu C., Yue R.C., Yang Y., Cui Y.Y., Yang L.F., Zhao D.M., Zhou X.M. (2016). AIM2 inhibits autophagy and IFN-β production during M. bovis infection. Oncotarget.

[73] Collins C.A., Cai H., Li T., Franco L.H., Li X.D., Nair V.R., Scharn C.R., Stamm L., Chen Z.J., Shiloh M.U. (2015). Cyclic GMP-AMP Synthase (cGAS) is an Innate Immune DNA Sensor for *Mycobacterium tuberculosis*. Cell Host Microbe.

[74] Lohoff M., Mak T.W. (2005). Roles of interferon-regulatory factors in T-helper-cell differentiation. Nat. Rev. Immunol..

[75] Mamane Y., Heylbroeck C., Genin P., Algarte M., Servant M.J., LePage C., DeLuca C., Kwon H., Lin R., Hiscott J. (1999). Interferon regulatory factors: The next generation. Gene.

[76] Taniguchi T., Takaoka A. (2001). A weak signal for strong responses: Interferon-α/β revisited. Nat. Rev. Mol. Cell Biol..

[77] Lin R., Heylbroeck C., Pitha P.M., Hiscott J. (1998). Virus-dependent phosphorylation of the IRF-3 transcription factor regulates nuclear translocation, transactivation potential, and roteasomemediated degradation. Mol. Cell Biol..

[78] Sato M., Tanaka N., Hata N., Oda E., Taniguchi T. (1998). Involvement of the IRF family transcription factor IRF-3 in virusinduced activation of the *IFN-β* gene. FEBS Lett..

[79] Sato M., Hata N., Asagiri M., Nakaya T., Taniguchi T., Tanaka N. (1998). Positive feedback regulation of type I IFN genes by the IFN-inducible transcription factor IRF-7. FEBS Lett..

[80] Marie I., Durbin J.E., Levy D.E. (1998). Differential viral induction of distinct interferon-α genes by positive feedback through interferon regulatory factor-7. EMBO J..

[81] Hata N., Sato M., Takaoka A., Asagiri M., Tanaka N., Taniguchi T. (2001). Constitutive IFN-α/β signal for efficient *IFN-α/β* gene induction by virus. Biochem. Biophys. Res. Commun..

[82] Honda K., Yanai H., Negishi H., Asagiri M., Sato M., Mizutani T., Shimada N., Ohba Y., Takaoka A., Yoshida N. (2005). IRF-7 is the master regulator of type-I interferon-dependent immune responses. Nature.

[83] Sato M., Suemori H., Hata N., Asagiri M., Ogasawara K., Nakao K., Nakaya T., Katsuki M., Noguchi S., Tanaka N. (2000). Distinct and essential roles of transcription factors IRF-3 and IRF-7 in response to viruses for *IFN-α/β* gene induction. Immunity.

[84] Erlandsson L., Blumenthal R., Eloranta M.L. (1998). Interferon-β is required for interferon-a production in mouse fibroblasts. Curr. Biol..

[85] Stockinger S., Reutterer B., Schaljo B., Schellack C., Brunner S., Materna T., Yamamoto M., Akira S., Taniguchi T., Murray P.J. (2004). IFN regulatory factor 3-dependent induction of type I IFNs by intracellular bacteria is mediated by a TLR- and Nod2-independent mechanism. J. Immunol..

[86] Ishii K.J., Coban C., Kato H., Takahashi K., Torii Y., Takeshita F., Ludwig H., Sutter G., Suzuki K., Hemmi H. (2006). A Toll-like receptor-independent antiviral response induced by doublestranded B-form DNA. Nat. Immunol..

[87] Stetson D.B., Medzhitov R. (2006). Recognition of cytosolic DNA activates an IRF3-dependent innate immune response. Immunity.

[88] Kawai T., Takeuchi O., Fujita T., Inoue J., Muhlradt P.F., Sato S., Hoshino K., Akira S. (2001). Lipopolysaccharide stimulates the MyD88-independent pathway and results in activation of IRF-3 and the expression of a subset of LPS inducible genes. J. Immunol..

[89] Doyle S., Vaidya S., O’Connell R., Dadgostar H., Dempsey P., Wu T., Rao G., Sun R., Haberland M., Modlin R. (2002). IRF3 mediates a TLR3/TLR4-specific antiviral gene program. Immunity.

[90] Kawai T., Adachi O., Ogawa T., Takeda K., Akira S. (1999). Unresponsiveness of MyD88-deficient mice to endotoxin. Immunity.

[91] Alexopoulou L., Holt A.C., Medzhitov R., Flavell R.A. (2001). Recognition of double-stranded RNA and activation of NF-κB by Toll-like receptor 3. Nature.

[92] Au W.C., Moore P.A., Lowther W., Juang Y.T., Pitha P.M. (1995). Identification of a member of the interferon regulatory factor family that binds to the interferon-stimulated response element and activates expression of interferon-induced genes. Proc. Nat. Acad. Sci. USA.

[93] Stadler M., Chelbi-Alix M.K., Koken M.H., Venturini L., Lee C., Saib A., Quignon F., Pelicano L., Guillemin M.C., Schindler C. (1995). Transcriptional induction of the PML growth suppressor gene by interferons is mediated through an ISRE and a GAS element. Oncogene.

[94] Nakaya T., Sato M., Hata N., Asagiri M., Suemori H., Noguchi S., Tanaka N., Taniguchi T. (2001). Gene induction pathways mediated by distinct IRFs during viral infection. Biochem. Biophys. Res. Commun..

[95] Yu Y., Wang S.E., Hayward G.S. (2005). The KSHV immediate early transcription factor RTA encodes ubiquitin E3 ligase activity that targets IRF7 for proteosome-mediated degradation. Immunity.

[96] Oeckinghaus A., Ghosh S. (2009). The NF-κB Family of Transcription Factors and Its Regulation. Cold Spring Harb. Perspect. Biol..

[97] Baldwin A.S. (1996). The NF-κB and IκB proteins: New discoveries and insights. Annu. Rev. Immunol..

[98] Akira S., Uematsu S., Takeuchi O. (2006). Pathogen recognition and innate immunity. Cell.

[99] Matsumoto M., Funami K., Tanabe M., Oshiumi H., Shingai M., Seto Y., Yamamoto A., Seya T. (2003). Subcellular localization of Toll-like receptor 3 in human dendritic cells. J. Immunol..

[100] Fitzgerald K.A., McWhirter S.M., Faia K.L., Rowe D.C., Latz E., Golenbock D.T., Coyle A.J., Liao S.M., Maniatis T. (2003). IKK epsilon and TBK1 are essential components of the IRF3 signaling pathway. Nat. Immunol..

[101] Perry A.K., Chow E.K., Goodnough J.B., Yeh W.C., Chen G. (2004). Differential requirement for TANK binding kinase-1 in type I interferon responses to toll-like receptor activation and viral infection. J. Exp. Med..

[102] Brasier A.R., Brasier A.R., García-Sastre A., Lemon S.M. (2008). The NF-κβ signaling network: Insights from systems approaches. Cellular Signaling and Innate Immune Responses to RNA Virus Infections.

[103] Li X., Kimbrel E.A., Kenan D.J., McDonnell D.P. (2002). Direct interactions between corepressors and coactivators permit the integration of nuclear receptor mediated repression and activation. Mol. Endocrinol..

[104] Leung T., Hoffmann A., Baltimore D. (2004). One nucleotide in a κB site can determine cofactor specificity for NF-κB dimers. Cell.

[105] Perissi V., Aggarwal A., Glass C.K., Rose D.W., Rosenfeld M.G. (2004). A core repressor/coactivator exchange complex required for transcriptional activation by nuclear receptors and other regulated transcription factors. Cell.

[106] Ogawa S., Lozach J., Pascua I.G., Tangirala R.K., Westin S. (2005). Molecular determinants of crosstalk between nuclear receptors and toll-like receptors. Cell.

[107] Theofilopoulos A.N., Baccala R., Beutler B., Kono D.H. (2005). Type I interferons (α/β) in immunity and autoimmunity. Annu. Rev. Immunol..

[108] Mancuso G., Midiri A., Biondo C., Beninati C., Zummo S., Galbo R., Tomasello F., Gambuzza M., Macrì G., Ruggeri A. (2007). Type I IFN signaling is crucial for host resistance against different species of pathogenic bacteria. J. Immunol..

[109] De Weerd N.A., Vivian J.P., Nguyen T.K., Mangan N.E., Gould J.A., Braniff S.J., Zaker-Tabrizi L., Fung K.Y., Forster S.C., Beddoe T. (2013). Structural basis of a unique interferon-β signaling axis mediated via the receptor IFNAR1. Nat. Immunol..

[110] Fusco D.N., Risac B.C., John S.P., Huang Y.W., Chin C.R., Xie T., Zhao H., Jilg N., Zhang L., Chevaliez S. (2013). A genetic screen identifies interferon-α effector genes required to suppress hepatitis C virus replication. Gastroenterology.

[111] Forster S. (2012). Interferon signatures in immune disorders and disease. Immunol. Cell Biol..

[112] Zhao H., Lin W., Chung R.T. (2012). A functional genomic screen reveals novel host genes that mediate interferon-α’s effects against hepatitis C virus. J. Hepatol..

[113] Meng Q.L., Liu F., Yang X.Y., Liu X.M., Zhang X., Zhang C., Zhang Z.D. (2014). Identification of latent tuberculosis infectionrelated microRNAs in human U937 macrophages expressing *Mycobacterium tuberculosis* Hsp16.3. BMC Microbiol..

[114] Schoggins J.W., MacDuff D.A., Imanaka N., Gainey M.D., Shrestha B., Eitson J.L., Mar K.B., Richardson R.B., Ratushny A.V., Litvak V. (2014). Pan-viral specificity of IFN-induced genes reveals new roles for cGAS in innate immunity. Nature.

[115] Rongvaux A., Jackson R., Harman C.C.D., Li T., West A.P., de Zoete M.R., Wu Y., Yordy B., Lakhani S.A., Kuan C.Y. (2014). Apoptotic caspases prevent the induction of type I interferons by mitochondrial DNA. Cell.

[116] Bogunovic D., Byun M., Durfee L.A., Abhyankar A., Sanal O., Mansouri D., Salem S., Radovanovic A., Grant V., Adimi P. (2012). Mycobacterial disease and impaired IFN-γ immunity in humans with inherited ISG15 deficiency. Science.

[117] Denis M. (1991). Recombinant murine β interferon enhances resistance of mice to systemic *Mycobacterium avium* infection. Infect. Immunol..

[118] Bloom C.I., Graham C.M., Berry M.P., Wilkinson K.A., Oni T., Rozakeas F., Xu Z., Rossello-Urgell J., Chaussabel D., Banchereau J. (2012). Detectable changes in the blood transcriptome are present after two weeks of antituberculosis therapy. PLoS ONE.

[119] Bouchonnet F., Boechat N., Bonay M., Hance A.J. (2002). α/β interferon impairs the ability of human macrophages to control growth of *Mycobacterium bovis* BCG. Infect. Immunol..

[120] O’Connell R.M., Saha S.K., Vaidya S.A., Bruhn K.W., Miranda G.A., Zarnegar B., Perry A.K., Nguyen B.O., Lane T.F., Taniguchi T. (2004). Type I interferon production enhances susceptibility to *Listeria* monocytogenes infection. J. Exp. Med..

[121] Manca C., Tsenova L., Bergtold A., Freeman S., Tovey M., Musser J.M., Barry C.E., Freedman V.H., Kaplan G. (2001). Virulence of a *Mycobacterium tuberculosis* clinical isolate in mice is determined by failure to induce Th1 type immunity and is associated with induction of IFN-α/β. Proc. Natl. Acad. Sci. USA.

[122] Antonelli L.R., Gigliotti R.A., Goncalves R., Roffe E., Cheever A.W., Bafica A. (2010). Intranasal Poly-IC treatment exacerbates tuberculosis in mice through the pulmonary recruitment of a pathogenpermissive monocyte/macrophage population. J. Clin. Investig..

[123] Dorhoi A., Yeremeev V., Kaufmann S.H.E. (2014). Type I IFN signaling triggers immunopathology in tuberculosis-susceptible mice by modulating lung phagocyte dynamics. Eur. J. Immunol..

[124] De Toledo-Pinto T.G., Ferreira A.B., Ribeiro-Alves M., Rodrigues L.S., Batista-Silva L.R., Silva B.J., Lemes R.M., Martinez A.N., Sandoval F.G., Alvarado-Arnez L.E. (2016). STING-dependent 2′-5′ oligoadenylate synthetase—Like production is required for intracellular *Mycobacterium leprae* survival. J. Infect. Dis..

[125] Mariotti S., Teloni R., Iona E., Fattorini L., Romagnoli G., Gagliardi M.C., Orefici G., Nisini R. (2004). *Mycobacterium tuberculosis* Diverts α Interferon-Induced Monocyte Differentiation from Dendritic Cells into Immuno-privileged Macrophage-Like Host Cells. Infect. Immun..

[126] Wilson E.B., Yamada D.H., Elsaesser H., Herskovitz J., Deng J., Cheng G., Aronow B.J., Karp C.L., Brooks D.G. (2013). Blockade of chronic type I interferon signaling to control persistent LCMV infection. Science.

[127] Kulpa D.A., Lawani M., Cooper A., Peretz Y., Ahlers J., Sekaly R.P. (2013). PD-1 co-inhibitory signals: The link between pathogenesis and protection. Semin. Immunol..

[128] Shi S., Blumenthal A., Hickey C.M., Gandotra S., Levy D., Ehrt S. (2005). Expression of many immunologically important genes in *Mycobacterium tuberculosis*-infected macrophages is independent of both TLR2 and TLR4 but dependent on IFN- αβ receptor and STAT1. J. Immunol..

[129] McNab F.W., Ewbank J., Howes A., Moreira-Teixeira L., Martirosyan A., Ghilardi N., Saraiva M., O’Garra A. (2014). Type I IFN induces IL-10 production in an IL-27-independent manner and blocks responsiveness to IFN-γ for production of IL-12 and bacterial killing in *Mycobacterium tuberculosis*-infected macrophages. J. Immunol..

[130] Giosue S., Casarini M., Alemanno L., Galluccio G., Mattia P., Pedicelli G., Rebek L., Bisetti A., Ameglio F. (1998). Effects of aerosolized interferon-α in patients with pulmonary tuberculosis. Am. J. Respir. Crit. Care Med..

[131] Giosuè S., Casarini M., Ameglio F., Zangrilli P., Palla M., Altieri A.M., Bisetti A. (2000). Aerosolized interferon-α treatment in patients with multi-drug-resistant pulmonary tuberculosis. Eur. Cytokine Netw..

[132] Cousens L.P., Peterson R., Hsu S., Dorner A., Altman J.D., Ahmed R., Biron C.A. (1999). Two roads diverged: Interferon α/β- and interleukin 12-mediated pathways in promoting T cell interferon α responses during viral infection. J. Exp. Med..

[133] Hermann P., Rubio M., Nakajima T., Delespesse G., Sarfati M. (1998). IFN-α priming of human monocytes differentially regulates gram-positive and gram-negative bacteria-induced IL-10 release and selectively enhances IL-12 p70, CD80, and MHC class I expression. J. Immunol..

[134] López-Collazo E., Hortelano S., Rojas A., Boscá L. (1998). Triggering of peritoneal macrophages with IFN-/ attenuates the expression of inducible nitric oxide synthase through a decrease in NF-B activation. J. Immunol..

[135] Philippot V., Yassir F., Balme B., Perrot H. (1996). Abcès sous-cutané à *Mycobacterium avium-intracellulare* après injections d’interféron α chez une malade traitée pour lymphome. Ann. Dermatol. Venereol..

[136] Toren A., Ackerstein A., Gazit D., Or R., Raveh D., Kupolovicz U., Engelhard D., Naglar A. (1996). Oral tuberculosis following autologous bone marrow transplantation for Hodgkin’s disease with interleukin-2 and α-interferon immunotherapy. Bone Marrow Transplant..

[137] Prabhakar S., Qiao Y., Hoshino Y., Weiden M., Canova A., Giacomini E., Coccia E., Pine R. (2003). Inhibition of Response to α Interferon by *Mycobacterium tuberculosis*. Infect. Immun..

[138] Ma J., Yang B., Yu S., Zhang Y., Zhang X., Lao S., Chen X., Li B., Wu C. (2014). Tuberculosis antigen-induced expression of IFN-α in tuberculosis patients inhibits production of IL-1β. FASEB J..

[139] Briken V., Sarah E.A., Shah S. (2013). *Mycobacterium tuberculosis* and the host cell inflammasome: A complex relationship. Front. Cell. Infect. Microbiol..

[140] Shin D.M., Jeon B.Y., Lee H.M., Jin H.S., Yuk J.M., Song C.H. (2010). *Mycobacterium tuberculosis* eis regulates autophagy, inflammation, and cell death through redox-dependent signaling. PLoS Pathog..

[141] Zullo A.J., Lee S. (2012). Mycobacterial induction of autophagy varies by species and occurs independently of mammalian target of rapamycin inhibition. J. Biol. Chem..

[142] Henry T., Brotcke A., Weiss D.S., Thompson L.J., Monack D.M. (2007). Type I interferon signaling is required for activation of the inflammasome during *Francisella* infection. J. Exp. Med..

[143] Jones J.W., Kayagaki N., Broz P., Henry T., Newton K., O’rourke K. (2010). Absent in melanoma 2 is required for innate immune recognition of *Francisella tularensis*. Proc. Natl. Acad. Sci. USA.

[144] Tsuchiya K., Hara H., Kawamura I., Nomura T., Yamamoto T., Daim S. (2010). Involvement of absent in melanoma 2 in inflammasome activation in macrophages infected with *Listeria monocytogenes*. J. Immunol..

[145] Fernandes-Alnemri T., Yu J.W., Juliana C., Solorzano L., Kang S., Wu J. (2010). The AIM 2 inflammasome is critical for innate immunity to *Francisella tularensis*. Nat. Immunol..

[146] Velmurugan K., Chen B., Miller J.L., Azogue S., Gurses S., Hsu T. (2007). *Mycobacterium tuberculosis* nuoG is a virulence gene that inhibits apoptosis of infected host cells. PLOS Pathog..

[147] Desvignes L., Wolf A.J., Ernst J.D. (2012). Dynamic roles of type I and type II interferons in early infection with *Mycobacterium tuberculosis*. J. Immunol..

[148] Guidotti L.G., Chisari F.V. (2006). Immunobiology and pathogenesis of viral hepatitis. Annu. Rev. Pathol..

[149] Su A.I., Pezacki J.P., Wodicka L., Brideau A.D., Supekova L., Thimme R., Wieland S., Bukh J., Purcell R.H., Schultz P.G. (2002). Genomic analysis of the host response to hepatitis C virus infection. Proc. Natl. Acad. Sci. USA.

[150] Imperiali F.G., Zaninoni A., La Maestra L., Tarsia P., Blasi F., Barcellini W. (2001). Increased *Mycobacterium tuberculosis* growth in HIV-1-infected human macrophages: Role of tumour necrosis factor-α. Clin. Exp. Immunol..

[151] Doherty T.M., Chougnet C., Schito M., Patterson B.K., Fox C., Shearer G.M., Englund G., Sher A. (1999). Infection of HIV-1 transgenic mice with *Mycobacterium avium* induces the expression of infectious virus selectively from a Mac-1-positive host cell population. J. Immunol..

